# The impact of the COVID-19 pandemic on lifestyle patterns: Does gender matter?

**DOI:** 10.3389/fpubh.2022.920694

**Published:** 2022-09-07

**Authors:** Naznin Sultana, Md. Asaduzzaman, Mahfuza Mubarak, Ismail Hosen, Mark Mohan Kaggwa, Firoj Al-Mamun, Mohammed A. Mamun

**Affiliations:** ^1^CHINTA Research Bangladesh, Savar, Bangladesh; ^2^Department of Public Health, North South University, Dhaka, Bangladesh; ^3^Department of Public Health Nutrition, Primeasia University, Dhaka, Bangladesh; ^4^Department of Public Health and Informatics, Jahangirnagar University, Savar, Bangladesh; ^5^African Centre for Suicide Prevention and Research, Mbarara, Uganda; ^6^Department of Psychiatry, Faculty of Medicine, Mbarara University of Science and Technology, Mbarara, Uganda; ^7^Department of Public Health, University of South Asia, Dhaka, Bangladesh

**Keywords:** COVID-19, lifestyle patterns, physical activity, substance use, prevalence, behavioral factors

## Abstract

**Background:**

The COVID-19 pandemic has significantly impacted individuals to deviate from normal lifestyle behaviors. But, there is a paucity of studies conducted in Bangladesh assessing how lifestyle patterns (i.e., smoking, drug use, physical exercise) have changed after the pandemic, which was investigated in this study.

**Methods:**

An online-based cross-sectional survey was conducted among a total of 756 Bangladeshi young adults between April 1 and 13, 2020. Lifestyle patterns data were collected based on two periods from the COVID-19 pandemic inception point in the country, (i) ‘1 year before’, and (ii) ‘1 year after’. Basic descriptive statistics (i.e., frequency and percentages) and Chi-square tests were performed to examine the associations of the independent variables in relation to lifestyle patterns.

**Results:**

A 0.2 and 4.7% reduction in smoking and physical exercise, respectively, was observed after the pandemic. But the prevalence of drug use was 1.5% before the COVID-19 pandemic, which rose to 1.9% during the pandemic; representing a 0.4% increment. The changes in lifestyle patterns before and during the COVID-19 pandemic was statistically significant only for physical exercise. Of the gender, male participants were more prevalent in smoking, drug use, and performing physical exercise in both periods.

**Conclusion:**

It is suggested to increase awareness concerning adverse effects of drug use and not performing physical exercise, where the gender-based focus is highly appreciated.

## Introduction

The virus, 2019-nCoV pneumonia, emerged from a Chinese fish market in Wuhan at the beginning of December 2019. Subsequently, the outbreak became a Public Health Emergency of International Concern, and later as a pandemic. The pandemic has soon become a global health issue affecting all aspects of human life. Due to the changes imposed by the pandemic, there is a massive impact on lifestyle (1, 2). For instance, Akter et al. found that there was a 4.4% increment in overweight after the pandemic inception in Bangladesh (30.5% ‘before’ the COVID-19 pandemic, to 34.9% ‘during’ the pandemic) (3). Such changes further impact mental health status, including suicidality (4). However, a Ugandan study based on a hospital registry found that 5.90% of adolescents had substance use disorders before the pandemic, which raise to 9.80% during the pandemic; but such change was not statistically significant (5). However, the impact of the pandemic on behavioral aspects is still an issue of consideration.

Behavioral health can be defined as an accumulation of physical and mental wellbeing where individuals play a crucial role in maintaining health and preventing disease and dysfunction (6). Healthy lifestyle behavior such as being free from substance use, performing regular physical exercise, etc., can often affect mental health. It can affect several factors such as emotional wellbeing, healthy behavioral adjustment, relative relief from anxiety and disabling symptoms, and the ability to build a meaningful relationship along with difficulties and challenges (7). However, lifestyle is viewed as a multidimensional construct encompassing a broader range of behaviors, including smoking, alcohol or substance abuse, stress management, social support, screen time, and digital technology usage (8–10). During the COVID-19 (Coronavirus disease) pandemic, people's lifestyles changed due to the implementation of different non-pharmaceutical measures such as social distancing, lockdowns, quarantine for suspected cases, and shutting down of educational institutions, and restricted all types of movement except emergency cases (11, 12). Consequently, sedentary behavior, abnormal sleeping patterns, smoking habits, physical inactivity, and consuming alcohol have been increased (13–15), which are the common risk factors for several non-communicable diseases, including mental health problems (16–19).

In the prior pandemic, such as SARS (Severe acute respiratory syndrome), evidence suggests an increment in drinking and smoking frequency compared to the period before the pandemic (20). Evidently, during the COVID-19 pandemic, several studies had reported changes in daily lifestyle behaviors (i.e., physical inactivity, smoking, and drug/substance use) (21, 22). For instance, consumption of recreational drugs has increased since the pandemic in the USA and Canada; that is, an increment of 23 and 16% for alcohol abuse and drug abuse among the prior pandemic consumers (23). On the other hand, more than 17 and 30% of Italian people reported increasing their alcohol and cigarette consumption, respectively, whereas many former consumers started reusing (21). Such increment is because stressed people during the lockdown time preferred to cope with emotional distress by unhealthy stress coping behaviors such as smoking and drug use; cross-national studies also support that (24). In addition, the pandemic was also responsible for changes in physical activity as well as eating behaviors due to the resulting confinement that ensured. For instance, a study conducted amongst Italians indicated that 56% had reduced the time devoted to physical activity during the pandemic, whereas inappropriate eating behavior such as increasing unhealthy food without increasing healthy ones had increased to 29.9% (21). Other studies also confirmed that both physically active or inactive people before the pandemic are less likely to perform physical activities; for example, 40.5 and 22.4% reduction among Canadian physically inactive and active people are reported after the pandemic inception (25).

In line with the situation, Bangladeshi people are anticipated to be at higher risk of performing unhealthy lifestyles, although there is a lack of evidence. For example, a study conducted during the early phase of the pandemic reported a prevalence of 37.9% physical inactivity (<600 MET–min/week), whereas a high level of sedentary behaviors (≥8 h/day) was also reported (20.9%) (26). Nevertheless, another study reported a reduction in physical activity at about 6.5% (4). Furthermore, a 15.9% increment in elderly's tobacco use after the inception of the pandemic was also found (27). Evidently, there is a need for further studies investigating the impact of the pandemic on unhealthy lifestyles for adopting appropriate policy and practice. Therefore, this study aimed to explore – (i) the prevalence of lifestyle patterns (smoking, drug use, and physical exercise) based on two time periods, 1 year before and 1 year after the pandemic inception in Bangladesh, (ii) the changes in these lifestyle patterns after the pandemic inception, and (iii) the factors influencing these lifestyle patterns.

## Methods

### Study procedure, participants, and ethics

A cross-sectional online survey was carried out among Bangladeshi young adults from April 1 to 13, 2020. Popular social media platforms (i.e., Facebook, WhatsApp) in Bangladesh were used to circulate the survey link. The criteria for participating in this study included being a Bangladeshi young adult resident. The data was collected using the snowball sampling technique to ensure maximum participation in the study. Approximately 782 data were collected, where a total of 756 data was used for final analysis after removing incomplete responses. The Helsinki Declaration 2013 was also considered in the study to ensure ethical aspects of the participants. Before conducting the study, an IRB approval was granted by the ethics committee at the Institute of Allergy and Clinical Immunology of Bangladesh (Reference: IRBIACIB/CEC/03202032). An online consent form was required for participating in the study.

### Sample size calculation

The sample size was calculated using the following formula


$$n=z^{2}pq/d^{2}\;$$

Where, *n* = sample size, *z* = 1.96 for 95% of confidence interval, *p* = prevalence, 50%; *q* = (1-p); *d* = 5% margin of error. In this way, the estimated sample size is 384. Considering a 10% non-response rate, the final estimated sample size was 424.

### Measures

#### Independent variables

A number of sociodemographic information such as age, gender, marital status, religion, educational qualification, socio-economic status (categorized based on 15,000 BDT intervals for higher, middle, and lower class), occupation, current residence status, etc. were collected. Participants were asked if they were suffering from any kind of comorbidities such as asthma, hypertension, heart disease, cardiovascular disease, cancer, diabetes, and others, based on a binary response. In addition, perceived self-rated health condition was assessed using a five- point Likert item (very good to very bad). Of the COVID-19 related issues, three questions with binary responses were asked. This included if (i) the participant themselves were infected with COVID-19, (ii) their family members/friends were infected with COVID-19, and (iii) any deaths occurred within family/friends due to COVID-19 infection.

#### Dependent variables

Unhealthy lifestyle patterns such as smoking, drug use, and physical exercise were considered in this study as the dependent variables. A total of three items with binary responses (yes/no) were included to assess the participants' lifestyle patterns based on two time periods (that is, 1 year before and 1 year after the COVID-19 pandemic inception in Bangladesh). Firstly, questions were asked about whether they were smokers and drug users (i) before the pandemic and (ii) during the pandemic (28). Again, if the participants performed physical exercise at least 30 min daily (i) before the pandemic and (ii) during the pandemic – they were asked (29–31).

### Statistical analysis

Before conducting the analysis, data were cleaned by Microsoft Excel 2019. Then data were analyzed using the Statistical Package for Social Science (SPSS) version 25. Descriptive statistics (i.e., percentage, frequency, mean & standard deviation) and inferential statistics (i.e., Chi-square test) were performed. A Chi-square test was performed to examine the associations between dependent and independent variables. Smoking, drug use, and physical inactivity were considered as the dependent variables for the ‘before’ and ‘during’ the COVID-19 pandemic, whereas sociodemographic variables and COVID-19 related factors were the predictors. Additionally, the overall changes in the lifestyle patterns (i.e., smoking, drug use, and physical exercise) considering the pre and post pandemic have been observed by the McNemar's test. A *p*-value of < 0.05 was set as statistically significant.

## Results

### Characteristics of the participants

Table 1 presents the characteristics of the participants. Most of them were male (59%), aged between 21 and 23 years (47.9%), unmarried (90.6%), and Muslim (89.4%). About 80% of the participants had more than secondary education, 35.7% were from a higher-class family, and 82.7% were students. Furthermore, higher participants were recorded from the urban region (64.8%), about 56.2% reported good health status, 15.5% reported very good health status, and 27.2% were suffering from comorbidities. Moreover, only 6% were infected with COVID-19, 42.9 and 12.8% of the participants reported that their family or friends were COVID-19 infected and died being infected with the virus, respectively (Table 1).

**Table 1:** Distribution of the sociodemographic variables of the respondents.

**Variables**	**Frequency (*n)***	**Percentage (%)**
**Age**		
≤20 years	228	30.2
21 to 23 years	362	47.9
≥24 years	166	22
**Gender**		
Female	310	41
Male	446	59
**Marital status**		
Unmarried	685	90.6
Married	71	9.4
**Religion**		
Muslim	676	89.4
Hindu	80	10.6
**Education level**		
Secondary	151	20
More than secondary	605	80
**Socio-economic status**		
Lower class	189	25
Middle class	237	31.3
Higher class	270	35.7
**Occupation**		
Students	625	82.7
Non-students	131	17.3
**Residence**		
Rural	266	35.2
Urban	490	64.8
**Perceived health status**		
Very good	117	15.5
Good	425	56.2
Neither good nor bad	184	24.3
Bad	27	3.6
Very bad	3	0.4
**Comorbidity status**		
Yes	206	27.2
No	550	72.8
**Personal COVID-19 infection**		
No	711	94
Yes	45	6
**Friends or family COVID-19 infection**		
No	432	57.1
Yes	324	42.9
**Friends or family COVID-19 death**		
No	659	87.2
Yes	97	12.8

### Prevalence rate of lifestyle patterns and its changes

The prevalence rates of smoking, drug use, and physical exercise are reported in Tables 2, 3. About 12.4 and 12.2% of the participants reported smoking before and during the pandemic, respectively, which means that there is a 0.2% reduction in smoking behavior (Table 2). In addition, there was a 0.4% increment in drug use after the pandemic inception (from 1.5 before to 1.9% during the pandemic) (Table 3), whereas physical exercise decreased by 4.7% (from 49.3 before to 54.0% during the pandemic) (Table 4). The changes were significant only in terms of physical exercise (*p* < 0.05); whereas non-significant results were found for both smoking and drug use (*p* > 0.05).

**Table 2:** Associations between the independent variables and smoking.

**Variables**	**Before COVID-19 pandemic**			**During COVID-19 pandemic**		
	**Yes (94, 12.4%)**	**No (662, 87.6%)**	**χ^2^ (*p-*value)**	**Yes (92, 12.2%)**	**No (664, 87.8%)**	**χ^2^ (*p-*value)**
**Sociodemographic information**						
**Age**						
≤20 years	19, 8.3	209, 91.7	5.514 (0.063)	16, 7	212, 93	8.172 **(0.017)**
21 to 23 years	49, 13.5	313, 86.5		53, 14.6	309, 85.4	
≥24 years	26, 15.7	140, 84.3		23, 13.9	143, 86.1	
**Gender**						
Female	4, 1.3	306, 98.7	59.931 **(<0.001)**	3, 1	307, 99	61.687 **(<0.001)**
Male	90, 20.2	356, 79.8		89, 20	357, 80	
**Marital status**						
Unmarried	87, 12.7	598, 87.3	0.477 (0.490)	84, 12.3	601, 87.7	0.06 (0.807)
Married	7, 9.9	64, 90.1		8, 11.3	63, 88.7	
**Religion**						
Muslim	84, 12.4	592, 87.6	<0.001 (0.985)	82, 12.1	594, 87.9	0.009 (0.924)
Hindu	10, 12.5	70, 87.5		10, 12.5	70, 87.5	
**Education level**						
Secondary	21, 13.9	130, 86.1	0.376 (0.540)	20, 13.2	131, 86.8	0.204 (0.651)
More than secondary	73, 12.1	532, 87.9		72, 11.9	533, 88.1	
**Socio-economic status**						
Lower class	19, 10.1	170, 89.9	2.140 (0.343)	21, 11.1	168, 88.9	1.067 (0.587)
Middle class	35, 14.8	202, 85.2		34, 14.3	203, 85.7	
Higher class	36, 13.3	234, 86.7		33, 12.2	237, 87.8	
**Occupation**						
Students	72, 11.5	553, 88.5	2.767 (0.096)	70, 11.2	555, 88.8	3.171 (0.075)
Non-students	22, 16.8	109, 83.2		22, 16.8	109, 83.2	
**Residence**						
Rural	30, 11.3	236, 88.7	0.503 (0.478)	31, 11.7	235, 88.3	0.102 (0.750)
Urban	64, 13.1	426, 86.9		61, 12.4	429, 87.6	
**Perceived health status**						
Very good	21, 17.9	96, 82.1	7.724 (0.102)	20, 17.1	97, 82.9	7.322 (0.120)
Good	43, 10.1	382, 89.9		42, 9.9	383, 90.1	
Neither good nor bad	28, 15.2	156, 84.8		28, 15.2	156, 84.8	
Bad	2, 7.4	25, 92.6		2, 7.4	25, 92.6	
Very bad	–	3, 100.0		–	3, 100	
**Comorbidity status**						
Yes	23, 11.2	183, 88.8	0.518 (0.419)	24, 11.7	182, 88.3	0.071 (0.789)
No	71, 12.9	479, 87.1		68, 12.4	482, 87.6	
**COVID-19 related information**						
**Personal COVID-19 infection**						
No	–	–	–	87, 12.2	624, 87.8	0.050 (0.823)
Yes	–	–		5, 11.1	40, 88.9	
**Friends or family COVID-19 infection**						
No	–	–	–	52, 12.0	380, 88.0	0.017 (0.898)
Yes	–	–		40, 12.3	284, 87.7	
**Friends or family COVID-19 death**						
No	–	–	–	85, 12.9	574, 87.1	2.554 (0.110)
Yes	–	–		7, 7.2	90, 92.8	

The bold values indicated that the results are significant in the test.

**Table 3:** Association between the independent variables and drug use.

**Variables**	**Before COVID-19 pandemic**			**During COVID-19 pandemic**		
	**Yes (11, 1.5%)**	**No (745, 98.5%)**	**χ^2^ (*p-*value)**	**Yes (14, 1.9%)**	**No (742, 98.1%)**	**χ^2^ (*p-*value)**
**Sociodemographic information**						
**Age**						
≤20 years	2, 0.9	226, 99.1	0.778 (0.678)	4, 1.8	224, 98.2	0.371 (0.831)
21 to 23 years	6, 1.7	356, 98.3		6, 1.7	356, 98.3	
≥24 years	3, 1.8	163, 98.2		4, 2.4	162, 97.6	
**Gender**						
Female	0, 0	310, 100	7.759 **(0.005)**	1, 0.3	309, 99.7	6.761 **(0.009)**
Male	11, 2.5	435, 97.5		13, 2.9	433, 97.1	
**Marital status**						
Unmarried	11, 1.6	674, 98.4	1.157 (0.282)	13, 1.9	672, 98.1	0.085 (0.771)
Married	0, 0	71, 100		1, 1.4	70, 98.6	
**Religion**						
Muslim	8, 1.2	668, 98.8	3.286 (0.070)	10, 1.5	666, 98.5	4.879 **(0.027)**
Hindu	3, 3.8	77, 96.3		4, 5.0	76, 95.0	
**Education level**						
Secondary	3, 2.0	148, 98.0	0.372 (0.542)	4, 2.6	147, 97.4	0.660 (0.417)
More than secondary	8, 1.3	597, 98.7		10, 1.7	595, 98.3	
**Socio-economic status**						
Lower class	4, 2.1	185, 97.9	0.749 (0.688)	6, 3.2	183, 96.8	2.419 (0.298)
Middle class	4, 1.7	233, 98.3		5, 2.1	232, 97.9	
Higher class	3, 1.1	267, 98.9		3, 1.1	267, 98.9	
**Occupation**						
Students	7, 1.1	618, 98.9	2.823 (0.093)	9, 1.4	616, 98.6	3.366 (0.067)
Non-students	4, 3.1	127, 96.9		5, 3.8	126, 96.2	
**Residence**						
Urban	4, 1.5	262, 98.5	0.007 (0.934)	6, 2.3	260, 97.7	0.368 (0.544)
Rural	7, 1.4	483, 98.6		8, 1.6	482, 98.4	
**Perceived health status**						
Very good	3, 2.6	114, 97.4	1.716 (0.788)	4, 3.4	113, 96.6	3.972 (0.410)
Good	5, 1.2	420, 98.8		5, 1.2	420, 98.8	
Neither good nor bad	3, 1.6	181, 98.4		5, 2.7	179, 97.3	
Bad	–	27, 100		–	27, 100.0	
Very bad	–	3, 100		–	3, 100	
**Comorbidity status**						
Yes	1, 0.5	205, 99.5	1.857 (0.173)	3, 1.5	203, 98.5	0.244 (0.622)
No	10, 1.8	540, 98.2		11, 2.0	539, 98.0	
**COVID-19 related information**						
**Personal COVID-19 infection**						
No	–	–	–	12, 1.7	699, 98.3	1.769 (0.183)
Yes	–	–		2, 4.4	43, 95.6	
**Friends or family COVID-19 infection**						
No	–	–	–	8, 1.9	424, 98.1	<0.001 (1.00)
Yes	–	–		6, 1.9	318, 98.1	
**Friends or family COVID-19 death**						
No	–	–	–	13, 2.0	646, 98.0	0.413 (0.521)
Yes	–	–		1, 1.0	96, 99.0	

The bold values indicated that the results are significant in the test.

**Table 4:** Association between the independent variables and physical exercise.

**Variables**	**Before COVID-19 pandemic**			**During COVID-19 pandemic**		
	**Yes (383, 50.7%)**	**No (373, 49.3%)**	**χ^2^ (*p-*value)**	**Yes (348, 46%)**	**No (408, 54%)**	**χ^2^ (*p-*value)**
**Sociodemographic information**						
**Age**						
≤ 20 years	112, 49.1	116, 50.9	1.491 (0.474)	104, 45.6	124, 54.4	1.901 (0.386)
21 to 23 years	180, 49.7	182, 50.3		160, 44.2	202, 55.8	
≥24 years	91, 54.8	75, 45.2		84, 50.6	82, 49.4	
**Gender**						
Female	133, 42.9	177, 57.1	12.653 **(<0.001)**	121, 39.0	189, 61.0	10.363 **(0.001)**
Male	250, 56.1	196, 43.9		227, 50.9	219, 49.1	
**Marital status**						
Unmarried	343, 50.1	342, 49.9	1.010 (0.315)	311, 45.4	374, 54.6	1.166 (0.280)
Married	40, 56.3	31, 43.7		37, 52.1	34, 47.9	
**Religion**						
Muslim	344, 50.9	332, 49.1	0.131 (0.718)	313, 46.3	363, 53.7	0.188 (0.665)
Hindu	39, 48.8	41, 51.2		35, 43.8	45, 56.3	
**Education level**						
Secondary	77, 51.0	74, 49.0	0.008 (0.927)	66, 43.7	85, 56.3	0.410 (0.522)
More than secondary	306, 50.6	299, 49.4		282, 46.6	323, 53.4	
**Socio-economic status**						
Lower class	120, 63.5	69, 36.5	13.352 **(0.001)**	114, 60.3	75, 39.7	19.249 **(<0.001)**
Middle class	110, 46.4	127, 53.6		106, 44.7	131, 55.3	
Higher class	135, 50.0	135, 50.0		108, 40.0	162, 60.0	
**Occupation**						
Student	306, 49.0	319, 51.0	4.177 **(0.041)**	282, 45.1	343, 54.9	1.207 (0.272)
Non-student	77, 58.8	54, 41.2		66, 50.4	65, 49.6	
**Residence**						
Urban	139, 52.3	127, 47.7	0.417 (0.518)	138, 51.9	128, 48.1	5.650 (0.017)
Rural	244, 49.8	246, 50.2		210, 42.9	280, 57.1	
**Perceived health status**						
Very good	66, 56.4	51, 43.6	10.235 **(0.037)**	62, 53.0	55, 47.0	24.560 **(<0.001)**
Good	227, 53.4	198, 46.6		218, 51.3	207, 48.7	
Neither good nor bad	80, 43.5	104, 56.5		58, 31.5	126, 68.5	
Bad	9, 33.3	18, 66.7		9, 33.3	18, 66.7	
Very bad	1, 33.3	2, 66.7		1, 33.3	2, 66.7	
**Comorbidity status**						
Yes	105, 51.0	101, 49.0	0.011 (0.917)	94, 45.6	112, 54.4	0.018 (0.892)
No	278, 50.5	272, 49.5		254, 46.2	296, 53.8	
**COVID-19 related information**						
**Personal COVID-19 infection**						
No	–	–	–	326, 45.9	385, 54.1	0.157 (0.692)
Yes	–	–		22, 48.9	23, 51.1	
**Friends or family COVID-19 infection**						
No	–	–	–	216, 50.0	216, 50.0	6.389 **(0.011)**
Yes	–	–		132, 40.7	192, 59.3	
**Friends or family COVID-19 death**						
No	–	–	–	303, 46.0	356, 54.0	0.006 (0.939)
Yes	–	–		45, 46.4	52, 53.6	

The bold values indicated that the results are significant in the test.

### Association between the explanatory variables and smoking

Table 2 presents the associations between the explanatory variables and smoking status. Participants aged ≥24 years reported a higher rate of smoking than participants aged ≤ 20 years and 21 to 23 years before the pandemic (*p* = 0.063); whereas participants aged between 21 and 23 years reported a higher rate of smoking during the pandemic than ≤ 20 years and ≥24 years (*p* = 0.017). Male participants reported a significantly higher tendency to smoke than their counterparts in both periods (*p* < 0.001). Despite these, no other variables were significantly associated with the smoking status (Table 2).

### Association between the explanatory variables and drug use

Table 3 presents the associations between the explanatory variables and drug use. The age group was not statistically significant with drug use at both times, but the gender was. More specifically, male participants were predominantly higher among the drug users both before (*p* = 0.005) and during (*p* = 0.009) the pandemic. Also, it is observed that 5% of the participants, Hindu in religious status during the pandemic, were more likely to be drug users than Muslims (1.5%; *p* = 0.027). In addition, drug use was more prevalent among the COVID-19 infected participants (*p* = 0.183) (Table 3).

### Association between the explanatory variables and physical exercise

Table 4 presents the associations between the explanatory variables and physical exercise. Gender showed a significant difference in terms of physical exercise. That is, female participants were less physically active than their male counterpart (42.9 vs. 56.1%, *p* < 0.001 for before pandemic; and 39 vs. 50.9%, *p* = 0.001 for during pandemic). Also, lower-class people were physically more active than others, showing a significant relationship before and during the pandemic. Participants who perceived their health status as very good reported more physical exercise than others in both periods (*p* < 0.05). In addition, these participants' friends and family who were infected with COVID-19 were less likely to be active in performing physical exercise during the pandemic (*p* < 0.05) (Table 4).

## Discussion

The COVID-19 pandemic-related movement restriction and confinement are essential to suppress the infection rate of the virus. Such a situation can be stressful and may affect the way of normal living, leading to prolonged stays at home. As a result, is it not surprising to observe changes in common lifestyle patterns such as eating habits, physical activity, substance use, etc. However, for the first time in Bangladesh, this study investigated the prevalence rates of smoking, drug use, and physical exercise ‘before’ and ‘during’ the pandemic and the factors associated with these lifestyle patterns in both periods. In addition, how the prevalence rate of these patterns has changed after the pandemic is also observed.

It was found that there was a 0.2% reduction in smoking status after the pandemic's inception, although the prevalence of drug use was reported to be increased by 0.4%. However, the changes were not significant based on pre and post-pandemic COVID-19 status. This finding is contradictory to the only prior Bangladeshi study that observed a 15.9% increment in tobacco use among the elderly (27); this may be due to the difference between the study population of the two studies (i.e., young adults are considered in the present study) as well as other factors such as study time for considering the change from the pandemic inception point. Similar to this study, some studies reported decreasing the use of tobacco products after the inception of the pandemic (e.g., 3.3% in Italy (13), 58.8% (32), despite most of the studies suggesting there is an increment in smoking and drug use (21, 23, 24, 27). However, an increasing trend of drug use after the pandemic was found (i.e., by 0.4 from 1.5 to 1.9%). Similarly, in the Netherlands, about 41.3% of the respondents reported using more cannabis, whereas 49.4% used it more often than before (33). Another study in the USA and Canada reported that about 16% of the respondents were using recreational drugs (23), whereas the increment rate was 17% in Italy (21).

As demonstrated in previous studies, physical inactivity can be influenced by lack of motivation, time availability, and restricted access to parks, dance, and fitness centers (22). The present study reported that physical activity was significantly reduced by 4.7%, which is consistent with the prior study conducted in Bangladesh, suggesting a 6.5% reduction in physical activity (i.e., 40.7% before the pandemic vs. 47.2% during the pandemic) (4). Similarly, a study from neighboring countries such as India found a 12.5% reduction (i.e., 38.5 and 50.5% before and during the pandemic) in not routinely 30 min moderate-intensity aerobic exercises or sports (22). Other studies confirmed the present findings, for example, 56% reduction in Italy (21), 40.5, and 22.4% reduction for these physically active and inactive people, respectively, before the pandemic in Canada (25).

All forms of drug abuse, including initiation, escalation of use, addiction, and relapse following abstinence, present in gender differences (34). Regarding gender and lifestyle patterns, the male gender was highly prone to tobacco and drug use at both times. Consistent with the prior Bangladeshi findings, 0.5 and 20.7% substance use, including smoking, drug, and alcohol use for females and males, was reported (28). A review of studies reported that males are at a 2–3 times higher risk of drug dependency, where such difference can be because of sociodemographic factors and biological factors. Sex chromosomes alone or by associating with gonadal hormones influence gender-based risk (34). During the COVID-19 pandemic, similar findings to this study were reported. For example, a higher prevalence of substance use behavior is reported among Chinese males compared with females (i.e., 32.7, 11.6, and 7.2% of hazardous, harmful, and dependent alcohol use vs. 24.9, 1.9, and 1.2%) (35). Similarly, drugs like cannabis use increased among the Netherlands population during the early period of the COVID-19 pandemic compared to before the lockdown period, where females were reportedly using more cannabis (i.e., 50.4% for females and 36.5% for males) (33).

Although males were found at a higher risk of substance use behaviors in this study, females reported performing less physical exercise. Such gender differences in performing physical activities can be determined by social determinants related to gender norms, social acceptability of exercise, cultural acceptance of females to go outside for exercise, etc. (36, 37). During the COVID-19 pandemic, the Bangladeshi males are reported to be 1.3 times more at risk of physically inactive than females (38.9 vs. 36.4%) (26), which is consistent with this study in both periods before and during the pandemic. According to a Spanish study, male participants had remarkably decreased vigorous physical activity than female participants (21% for females and 9% for males), whereas it was 8.2 and 11% for moderate physical activities (38).

With respect to other sociodemographic factors of this study, young participants were found to be at more risk of being smokers after the pandemic's inception. But the other study reported opposite findings; for example, a 3.3 times higher risk of smoking during the pandemic was found among the Bangladeshi elderlies aged 60–69 years compared to 70 and above (27). Another study reported that physical inactivity was higher in upper-class people (47.5%) in comparison to lower and middle classes (23 and 35.9%) (26), which was the same in this study. People of the higher class are at more risk of performing less physical exercise; this may be due to avoiding social interaction in fear of COVID-19 infection and being confined as they have more financial support from other groups. In addition, the participants with poor self-rated health status were less likely to perform physical exercise. As well as having a history of COVID-19 infection in the participants' family or friend circles, they were at a higher likelihood of physical inactivity, which can be because of psychological distress turned by their COVID-19 suffering hindering performing physical exercise.

The study, for the first time, assesses (i) the prevalence rates of lifestyle patterns based on before and during the COIVD-19 pandemic, (ii) the factors associated with such rate, and (iii) the changes in lifestyle patterns prevalence rate after the pandemic. As an exploratory study, the findings reported in this study could add value to implicate further study and direct policymaking. However, it is worth mentioning that this study's findings can be limited because its study nature is cross-sectional, where causality can be inferred. Secondly, an online survey may involve selection bias, information bias, and also memory recall bias. Thirdly, the study involved the country's young adults, which can be limited because of not involve the general population. Fourthly, this study strength would be better if it was longitudinal in nature to assess such data types and avoid memory recall bias and collected data by repeatedly over a longer period of time. Finally, diet modification related factors were not considered for this study, which is one of the major factor of lifestyle related issues.

## Conclusions

Overall, this study demonstrates how the COVID-19 pandemic affects the lifestyle patterns of Bangladeshi young adults. Some of the baseline data with respect to the pandemic effect is provided, showing that the rate of smoking and physical activity was reduced during the COVID-19 pandemic compared to the before pandemic, although an inverse finding was reported for drug use and such changes were significant only for physical exercise. Male participants were more prevalent than females to be involved in unhealthy lifestyles like smoking and drug use, although females performed less physical exercise. Therefore, increasing awareness concerning the adverse effects of drug use and not performing physical exercise is suggested, where the gender-based focus is highly appreciated. Because it would be more effective to pay more attention to males while implementing any programs related to the adverse effect of drug use, whereas females can be motivated and provided familial and social support that includes ensuring available time and place for exercise to increase their participation in physical activities, which might prevent cardiovascular disease related complications as suggested by pervious literatures (39–41).

## Data availability statement

The raw data supporting the conclusions of this article will be made available by the authors, without undue reservation.

## Ethics statement

The studies involving human participants were reviewed and approved by Institute of Allergy and Clinical Immunology of Bangladesh. The patients/participants provided their written informed consent to participate in this study.

## Author contributions

MMa planned the study. NS and FA-M analyzed the data and contributed to data interpretation. NS, MA, FA-M, and MMa wrote the first draft. Critical review and edits were done by all authors, especially MMu and MMa. All authors partook in project implementation and management. All authors contributed to the article and approved the submitted version.

## Conflict of interest

The authors declare that the research was conducted in the absence of any commercial or financial relationships that could be construed as a potential conflict of interest. The reviewer SD declared a shared affiliation with the authors MMu, IH, FA-M, and MMa to the handling editor at the time of the review.

## Publisher's note

All claims expressed in this article are solely those of the authors and do not necessarily represent those of their affiliated organizations, or those of the publisher, the editors and the reviewers. Any product that may be evaluated in this article, or claim that may be made by its manufacturer, is not guaranteed or endorsed by the publisher.

## Abbreviations

COVID-19: Coronavirus disease 2019
SARS: severe acute respiratory syndrome
MET: Min per week
SPSS: Statistical Software for Social Science.
